# Mass drug administration for the prevention human strongyloidiasis should consider concomitant treatment of dogs

**DOI:** 10.1371/journal.pntd.0005735

**Published:** 2017-08-24

**Authors:** Meruyert Beknazarova, Harriet Whiley, Kirstin Ross

**Affiliations:** Department of Environmental Health, Flinders University, Adelaide, South Australia, Australia; University of Pennsylvania, UNITED STATES

*Strongyloides stercoralis* is the causative agent of strongyloidiasis, which affects more than 300 million people worldwide [1]. Canine strongyloidiasis is also caused by the same species, *S*. *stercoralis* of an animal origin with up to 50% worldwide prevalence [2–5]. It is known that human *S*. *stercoralis* strains can infect dogs in laboratory settings (dogs have to be immunosuppressed to maintain infection) [6–8]. Increasing evidence suggests that *S*. *stercoralis* could be a potential zoonotic pathogen [2, 5, 9–11].

A study by Goncalves et al. [9] examined 181 kennels and 11 dog keepers responsible for kennel cleaning in the southeastern region of Brazil. The serological analysis (ELISA) found that 24.3% (44 out of 181) and 33% (3 out of 9) of dogs and humans were infected with *S*. *stercoralis*, respectively [9]. In another study, stool examination identified rhabditiform larvae in the feces of an animal handler. Examination of his wife and his domesticated pet dog found no larvae present in their stools. However, one-third of the dogs under his charge had *S*. *stercoralis* larvae in their feces [10]. Another recent study identified *S*. *stercoralis* in dog using the *S*. *stercoralis* species-specific primers and probes commonly used to identify human *S*. *stercoralis* identification [12]. These studies suggest that dogs may play a role in human strongyloidiasis and/or that humans may play a role in canine strongyloidiasis.

There are a few studies describing the role of dogs in the spread of human strongyloidiasis. A study from Anima Islands, Japan, assessed more than 600 humans and their dogs and found no strongyloidiasis cross-contamination [13]. However, further studies are needed to explore the effect of differing interactions and behaviours between humans and dogs in different communities and cultures.

Genetic studies are useful for exploring the differences between dog and human *S*. *stercoralis* strains and provide insight into the potential for cross infection. The whole genome *S*. *stercoralis* sequence (accession number PRJEB528) described by Hunt et al. [14] was from a canine fecal sample. There are 2 regions of *S*. *stercoralis* that have been of a particular interest for nucleotide sequencing. Hyper-variable regions ([HVR] I-IV) in 18s ribosomal DNA and a cytochrome c-oxidase subunit 1 gene (cox1) region in mitochondrial DNA (mtDNA) are generally considered to be inter and intraspecific and are used to examine *S*. *stercoralis* populations of different geographic locations or hosts [15, 16]. A study by Hasegawa et al. [15] evaluated the HVR-I-IV regions among different species of *Strongyloides*. They found that for the HVR-I-III regions, multiple species frequently shared the same sequence. In HVR-I they describe minor sequence variations within *S*. *stercoralis* sampled from different hosts and locations. These differences, however, did not indicate the host of origin of the particular worm. HVR-IV appeared more species-specific, but did not show intra-specific variations in some *Strongyloides* spp., including *S*. *stercoralis*. In a following study Hasegawa et al. [16] sequenced the HVR-IV and the mtDNA cox1 gene in *S*. *stercoralis* and *S*. *fuelleborni* isolated from different hosts (humans, dogs, apes, and monkeys) and from different geographical locations. The HVR-IV region was again found species-specific. In the study by Hasegawa et al. [16], the authors described 1 within species polymorphism, which, however, occurred within the worms isolated from humans and, therefore, provided no indication of a separation of human and dog derived *S*. *stercoralis*. In contrast to this, in their earlier study, Hasegawa et al. [15] had noticed that based on a preliminary genetic analysis of the mtDNA, dog-derived *S*. *stercoralis* appeared phylogenetically distant from those of primate (including human) derived *S*. *stercoralis*. In the second study, [16] the mtDNA cox1 gene was found to be more conserved within *S*. *stercoralis* compared to *S*. *fuelleborni*. Nevertheless, based on the cox1 nucleotide sequences, *S*. *stercoralis* from dogs appeared phylogenetically separated from those isolated from humans. Further, there was 1 amino acid substitution identified, which consistently separated the admittedly rather small number of dog derived *S*. *stercoralis* from human derived *S*. *stercoralis* [16]. Genetic studies of human *S*. *stercoralis* from different geographic zones suggested that climatic conditions, such as temperature and moisture, may coincide with genetic changes [17–19]. More research examining the DNA sequences of dog and human *S*. *stercoralis* has to be done in order to improve our understanding of animal *S*. *stercoralis* infectivity to humans.

Presently, there is limited evidence regarding the role of dogs in human strongyloidiasis; however, there is enough to suggest that further research is needed to investigate this potential route of infection. Dogs carrying *S*. *stercoralis* in communities could possibly explain the limitations of previous Mass drug administration (MDA) programs; this is another area requiring further research. A study by Kearns et al. [20] investigated the prevalence of human strongyloidiasis and scabies in remote Australian Aboriginal communities to evaluate the efficacy of ivermectin MDA. The study demonstrated that ivermectin MDA reduced prevalence but failed to eliminate strongyloidiasis and scabies in the community [20]. Reappearance of strongyloidiasis could potentially be due to helminth resistance development or reinfection from environmental reservoirs (such as dogs, soil, etc.). Control of environmental reservoirs would also reduce reliance on MDA targeting humans as *S*. *stercoralis* ability to autoinfect in humans can compromise the success of the MDA, leading to potential helminth resistance development to a drug [21]. However, this would not be a concern for MDA of dogs as *S*. *stercoralis* tend to lose autoinfection ability in healthy dogs [5, 22].

Anecdotally, animal management strategies are already being undertaken in many remote communities, and as such, concurrent treatment with anthelmintic drugs would minimise costs. Mass vaccination of dogs, including oral drug treatment targeting stray dogs, have been successfully practiced over the years to significantly decrease the rabies prevalence among humans [23, 24]. The rabies elimination model has been estimated to cost between US$2 to US$5 for a single dog vaccination, suggesting that treatment of dogs presents an economically suitable option [24, 25]. Drug treatment of water buffaloes is another successful example of MDA when applied to a potential animal reservoir to control human schistosomiasis [26]. Future MDA programs should consider treating both humans and dogs to fight strongyloidiasis. Given that the relative cost of treating dogs is low, this potentially could provide a low cost and low-risk mechanisms to reduce the risk of reinfection.
